# Clinical outcomes and factors associated with mortality among adults admitted to the intensive care unit at Hawassa University Comprehensive Specialized Hospital, Ethiopia: retrospective chart review

**DOI:** 10.1186/s12871-025-03603-z

**Published:** 2026-01-16

**Authors:** Mulugeta Edao Shate, Robel Mesfin, Wondimagegn Genaneh, Bikila Lencha

**Affiliations:** 1 Department of Nursing, School of Medicine and Health Sciences, Hawassa University, Hawassa, Ethiopia; 2 Department of Anesthesia, School of Medicine and Health Science, College of Health Sciences, Dilla University, Dilla, Ethiopia; 3 Department of Nursing, Wolaita Sodo University, Sodo, Ethiopia; 4 Department of Epidemiology, School of Public Health, College of Health Sciences, Madda Walabu University, Shashemene, Ethiopia

**Keywords:** Intensive care unit, Adult, Mortality, Factors associated, Ethiopia

## Abstract

**Background:**

Intensive Care Units manage patients with life-threatening conditions. Despite all efforts, mortality in these units remains high in many low-resource settings. There is limited evidence on the outcomes and Factors Associated with mortality among adult patients admitted to intensive care in the Sidama region of Ethiopia.

**Objective:**

To assess clinical outcomes and Factors Associated with Mortality among Adults Admitted to the Intensive Care Unit at Hawassa University Comprehensive Specialized Hospital, Ethiopia.

**Methods and materials:**

A facility-based retrospective chart review was conducted among adult patients admitted to the ICU of Hawassa University Comprehensive Specialized Hospital between 1 January 2022 and 10 April 2024. Data were extracted from 401 patient charts using a structured checklist. The study participants’ cards were selected consecutively using a list of their medical record numbers. The collected data were checked for completeness and consistency. The coded data were checked and entered into the EPI data version 4.6. The data were then exported into SPSS version 26 for analysis. Bivariate and multivariable logistic regression analyses were performed to identify factors associated with ICU mortality. Variables with a *p*-value < 0.05 were considered significant.

**Results:**

The overall intensive care unit mortality was 28% (95% CI: 23.5–32.5). In the multivariate analysis, mortality was associated with mechanical ventilation requirements in patients (AOR = 5.71, 95% CI: 1.49–21.8) and unconscious mental status at admission (AOR = 4.76, 95% CI: 1.38–16.44). Patients who stayed less than four days in ICU are two times more likely to die than those who survived (AOR = 2.10, 95% CI: 1.25–3.53).

**Conclusion and recommendation:**

In this study, Mortality among adult patients admitted to the ICU was high. Mechanical ventilation, impaired level of consciousness at admission, and shorter ICU length of stay were factors associated with increased mortality. These findings highlight the need for improved early identification, stabilization, and management of critically ill patients in resource-limited settings.

## Introduction

An Intensive Care Unit (ICU) is a hospital unit dedicated to managing and monitoring patients with life-threatening conditions, which is specially staffed and equipped separately and is self-contained. It has special expertise and support equipment for vital functions and uses the skills of medical nurses and other professionals trained in addressing these problems [1]. Globally, ICU mortality varies substantially across regions. In developed countries such as North America, Oceania, Asia, and Europe, ICU mortality is 9.3%, 10.3%, 13.7%, and 18.7%, respectively. It is found to be 21.7% and 26.2% in the rest of the world, represented by South America and the Middle East [2, 3]. Mortality rates are usually higher in poor and middle-income countries. Recent audits conducted in sub-Saharan African regions reveal that mortality rates in the ICU were between 30% and 50%, due to late presentation, lack of critical-care equipment, shortfall in access to vital drugs, and absence of a well-accepted criterion of admission to the ICU [4, 5].

In East Africa, high ICU mortality has been reported in Kenya (40.1%) [6], Uganda (40–46%) [7], and Tanzania (41%) [8]. Abnormal vital signs, elderly patients, patients on mechanical ventilation, acute respiratory failure, readmission in intensive care, length of stay in intensive care, and sepsis are risk factors for mortality in the intensive care unit [4, 9].

In Ethiopia, critical care services are expanding rapidly, but important resource limitations persist. The first ICU opened over three decades ago at Tikur Anbessa Hospital [10], and since then, several public hospitals have followed suit, creating their own ICUs. However, most continue to operate with a limited number of beds, a shortage of ventilators, and a scarcity of trained critical-care providers [4, 11]. Previous studies conducted in Ethiopia reported high ICU mortality: 39% in Addis Ababa [11], 46.4% in Hossana [12], and 27% in Tigray [13]. Despite the notable advancements in critical care services driven by technological innovation and new scientific developments that enhance treatment outcomes for critically ill patients worldwide, the progression of critical care service delivery in resource-limited environments remains significantly behind, resulting in persistently higher mortality rates [4, 14].

In spite of a high demand for critical care services, only limited evidence is available from the ICU of Hawassa University Comprehensive Specialized Hospital (HUCSH) regarding the types of patients most frequently admitted, whether emergency medical, emergency surgical, trauma, or gynecologic cases. Moreover, little is known about which groups of patients consume the most ICU resources, their mortality rates, or to what extent protracted ICU stay increases patient costs. Understanding Clinical Outcomes and Predictors of Mortality among Adults Admitted is very important for effective resource planning and for the improvement of the hospital’s response to the most common and severe medical and surgical conditions in the population it serves. Thus, this study assessed the Clinical Outcomes and Factors Associated with Mortality among Adults Admitted to the Intensive Care Unit of HUCSH, Hawassa, and Sidama Region, Ethiopia.

## Methodology

### Study design, area, and period

This study employed a facility-based retrospective chart review conducted at Hawassa University Comprehensive Specialized Hospital (HUCSH), a tertiary referral hospital located in Hawassa City, Sidama Region, Ethiopia. This hospital offers adult intensive care services and serves as one of the key referring centers for patients with severe illnesses from the southern parts of Ethiopia. The hospital charts of adult patients who were admitted to intensive care units (ICU) between 1 January 2022 and 10 April 2024 were considered for review.

### Source of population

All recorded charts of adult patients admitted to the ICU of HUCSH hospital.

### Study population

Selected charts of adult patients admitted to the ICU of HUCSH from January 1, 2022, to April 10, 2024. The study population included adult patients whose medical records met the eligibility criteria.

### Inclusion and exclusion criteria

#### Inclusion criteria

All adult patients aged 18 years or older who were admitted to the ICU during the study period and had documented admission diagnosis, clinical information, and ICU discharge outcomes were included.

#### Exclusion criteria

Medical records with incomplete or missing key information, such as diagnosis, vital signs, ICU outcome, or length of stay, were excluded from the study.

### Sample size determination

The sample size is determined for assessment outcomes of patients admitted to the adult ICU using the formula of single population proportion of *P* = 46.42%, which is taken from research done in Clinical outcomes of patients admitted to ICU of Nigist Eleni Mohammed Memorial Hospital of Hosanna, Southern Ethiopia [12].

$$\begin{aligned} &\mathrm P=\;46.42\%\;\mathrm{with}\;5\%\;\mathrm{margin}\;\mathrm{of}\;\mathrm{erroor}\; \\& \mathrm n=\;\left(1.96\right)\;2\ast\left(0.46\right)\ast\left(1-0.46\right)/\left(0.05\right) \\&2=382 \end{aligned}$$

382 Based on the above assumption, the sample size was 382.

The calculated total sample size (*n*), including a 5% contingency for incomplete charts, was 401. The patients’ charts were reviewed at the Adult ICU in Hawassa Specialized Hospital.

Where *n* = sample size, Z = standard normal deviation, *P* = population proportion, and d = margin of error.

### Sampling technique

A consecutive sampling method was used, where all eligible charts of adult patients admitted to the ICU of HUCSH from January 1, 2022, to January 1, 2024, were included until the sample size was achieved.

### Study variables

####  Dependent variables

Clinical Outcomes (Survivors vs. Non-survivors) of patients admitted to the ICU in HUCSH.

#### Independent variables

socio-demographic data (Age, gender, residence area), clinical diagnosis at admission, presence of comorbid illness, source of referral, frequency and category of admission, vital signs at admission, intervention during ICU stay, and length of stay in the AICU.

### Operational definitions

 Intensive Care Unit Mortality (ICU) is death occurring during ICU admission [15].

 Incomplete information is the absence of all the necessary information on patients based on the standard formats attached in annex [13].

 Length of ICU Stay is the total number of days a patient remained in the ICU from admission until discharge, death, or transfer. A cut-off of < 4 days vs. ≥ 4 days was used based on previous studies evaluating early ICU mortality [16].

 Mechanical Ventilation is the use of invasive ventilator support via endotracheal tube or tracheostomy [17].

 Mental Status is categorized using GCS: Conscious (13–15), Confused (9–12), Unconscious (≤ 8) [18].

 Vital signs at ICU admission classified based on international standards: For pulse, Tachycardia: Pulse rate > 100 bpm, and Normal pulse: 60–100 bpm. For respirations, Bradypnea: RR < 12 breaths/min, and Tachypnea: RR > 20 breaths/min [19].

### Data collection procedure

The information was recorded on a structured data extraction checklist format, adapted from similar studies [12]. Based on the elements intended to be studied. The study data contains socio-demographic data of the study population, the average length of ICU stay, vital signs during admission, causes of ICU admission, and outcomes, including alive and death for each diagnosis. The data were extracted by trained 5 BSc Nurses. The data of patients were extracted from the patients’ registration books and cards admitted to the adult ICU.

### Data processing and analysis

After data extraction, the principal investigator checked the data for completeness and consistency. The coded data were checked and entered into the EPI data version 4.6. Then the clean data were exported and analyzed using the Statistical Package for Social Sciences (SPSS) version 26. Descriptive statistics were used to summarize patient characteristics and clinical variables. Categorical variables were presented as frequencies and percentages. Continuous variables were assessed for normality using visual inspection of histograms and the Shapiro–Wilk test. As continuous variables were not normally distributed, they were summarized using medians and interquartile ranges (IQRs). The fitness of logistic regression models was assessed using the Hosmer-Lemeshow test, and its *p*-value is 0.482. A Multicollinearity test was carried out to see the correlation between each independent variable, using the Variance Inflation Factor (VIF), and a tolerance test, and there were no variables that did not meet the criteria.

Finally, all variables with a *p*-value < 0.25 in the bivariable analysis were included in the initial multivariable logistic regression model. A backward stepwise selection method was applied to obtain a parsimonious final model and to identify independent factors associated with ICU Mortality. Adjusted odds ratios (AOR) with 95% confidence intervals (CI) were reported, and statistical significance was set at *p* < 0.05.

### Ethical consideration

Ethical clearance was obtained from Madda Walabu University, Shashemene Campus, Department of Public Health Institutional Research Ethical Review Committee (IRERC) (Ref. No: IRERC/013/24). Permission to conduct the study was secured from the administration of Hawassa University Comprehensive Specialized Hospital (HUCSH).

As this study was conducted using retrospective data extracted from medical records and did not include any direct contact with patients, informed consent was exempted by the Institutional Review Board. Confidentiality of data was strictly maintained by de-identifying all patient data before analysis. This study was performed in accordance with the ethical guidelines of the Declaration of Helsinki.

## Result

### Socio-demographic characteristics of the participants

Between January 1, 2022, and April 10, 2024, a total of 882 patients were admitted to the ICU. Of these, only 401 cards had complete data available for review. Continuous variables were assessed for normality using visual inspection of histograms and the Shapiro–Wilk test. Age was not normally distributed and is therefore summarized using the median and interquartile range. The median age of patients was 50 years (IQR: 41–61). The largest proportion of patients was aged 50–59 years (36.9%). More than half of the patients were female (56.1%), and 51.6% resided in urban areas. Nearly half of the patients were admitted from the medical ward (47.6%), followed by the emergency department (28.7%). Additional socio-demographic characteristics are presented in Table 1.

**Table 1:** Socio-demographic characteristics of adult patients admitted to the ICU at Hawassa University Comprehensive Specialized Hospital, Ethiopia (*n* = 401)

Characteristics	Category	Frequency	Percent
Age	< 49	135	33.7
	50–59	148	36.9
	≥ 60	118	29.4
	Median (IQR)	50 (41–61)	
Sex	male	176	43.9
	female	225	56.1
Residence	urban	207	51.6
	rural	194	48.4
Source of admission	Medical ward	191	47.6
	Emergency department	115	28.7
	Surgical ward	42	10.5
	Gynecology and Obstetrics Ward	53	13.2
Category of admission	Emergency medical patients	271	67.6
	Emergency surgical patients	130	32.4
Frequency of admission	new admission	377	94.0
	readmission	24	6.0

### Clinical characteristics of the disease

A total of 401 patient charts were included in the analysis. The median ICU stay was three days, but the majority (66.6%) had prolonged stays of more than four days, and one-third of patients (33.4%) stayed in the ICU for less than four days. The most common system-based diagnoses during ICU were respiratory diseases (23.2%), endocrine disorders (18.2%), neurologic diseases (16.2%), and cardiovascular conditions (17.5%). The main causes of death were acute respiratory distress syndrome (ARDS) 86 (21.4%), followed by shock 77 (19.2%), stroke 47(11.7%), Diabetic Ketoacidosis (DKA) 40(10.0%), and traumatic brain injury (TBI) 37 (9.7%) (Table 2).

**Table 2:** Clinical characteristics of adult patients admitted to the ICU at Hawassa University Comprehensive Specialized Hospital, Ethiopia (*n* = 401)

Characteristics	Category	Frequency	Percent
Length of ICU stay	More than four days	267	66.6
	less than four days	134	33.4
	Median (INQ)	3 (2–6)	
Common system-based diagnoses during ICU admission	Cardiovascular disease	70	17.5
	Respiratory disease	93	23.2
	Trauma	42	10.5
	Infectious disease	25	6.2
	Neurologic disease	65	16.2
	Endocrine disease	73	18.2
	Renal disease	19	4.7
	Hematologic disease	11	2.7
	Miscellaneous conditions	3	0.7
Common Causes of Death in Patients Admitted to the ICU	Diabetic Ketoacidosis	40	10.0
	ARDS	86	21.4
	CHF	36	9.0
	HIV	36	9.0
	Myocardial infarction (MI)	26	6.5
	Shock	77	19.2
	pneumonia	10	2.5
	Stroke	47	11.7
	TBI	37	9.7

*Abbreviation*: *TBI *traumatic brain injury, *HIV *human immunodeficiency virus, *ARDS* acute respiratory distress syndrome

### Vital signs of patients during ICU admission

Regarding the vital signs of patients during ICU admission, the majority of patients had unstable records. Two hundred ninety (72.3%) patients had high pulse rates, and 58 (14.5%) patients had a normal range pulse rate (60–100 beats per minute). Concerning respiratory rates, Patients had normal respiratory rates, 260 (64.8%), and 141(35.2%) patients were fast breathing or respiratory depression (Table 3).

**Table 3:** Distribution of admission vital signs for patients admitted to the ICU of Hawassa University Comprehensive Specialized Hospital, Ethiopia, 2024

Characteristics	Category	Frequency	Percent
Pulse rate (beats per minute)	< 60	58	14.5
	60–100	53	13.2
	> 100	290	72.3
Respiratory rate (breath per minute)	12–20	260	64.8
	> 20	141	35.2
Temperature	< 36. 5	257	64.1
	36.5–37.5	73	18.2
	> 37.5	71	17.7
oxygen saturation(SPO2)	< 90%	330	82.3
	90%−100%	71	17.7
GCS	Conscious (GCS 13–15)	134	33.4
	Confused (GCS 9–12)	152	37.9
	Unconscious (GCS 3–8).	115	28.7

### Clinical management of patients during intensive care unit stay

During the intensive care unit admission in the Intensive Care Unit of the Hawassa University Comprehensive Specialized Hospital in 2024, most of the patients did not require mechanical ventilator support, with 389 patients (97.0%) not receiving assisted breathing and only 12 patients (3.0%) receiving mechanical ventilation support. Vasoactive support was provided to 47 patients (11.7%), and the vast majority of patients, that is, 354 patients (88.3%), did not need any support with vasoactive agents. Antibiotic therapy was also used extensively, with 322 patients (80.3%) receiving antibiotic therapy and 79 patients (19.7%) not receiving antibiotic therapy (Table 4).

**Table 4:** Clinical management of adult patients admitted to the ICU at Hawassa University Comprehensive Specialized Hospital, Ethiopia, 2024

Characteristics	Category	Frequency	Percent
Mechanical ventilation	no	389	97.0
	yes	12	3.0
Use of a vasopressor	yes	47	11.7
	no	354	88.3
Use antibiotic	yes	322	80.3
	no	79	19.7

### Outcome during discharge

**Fig. 1:**
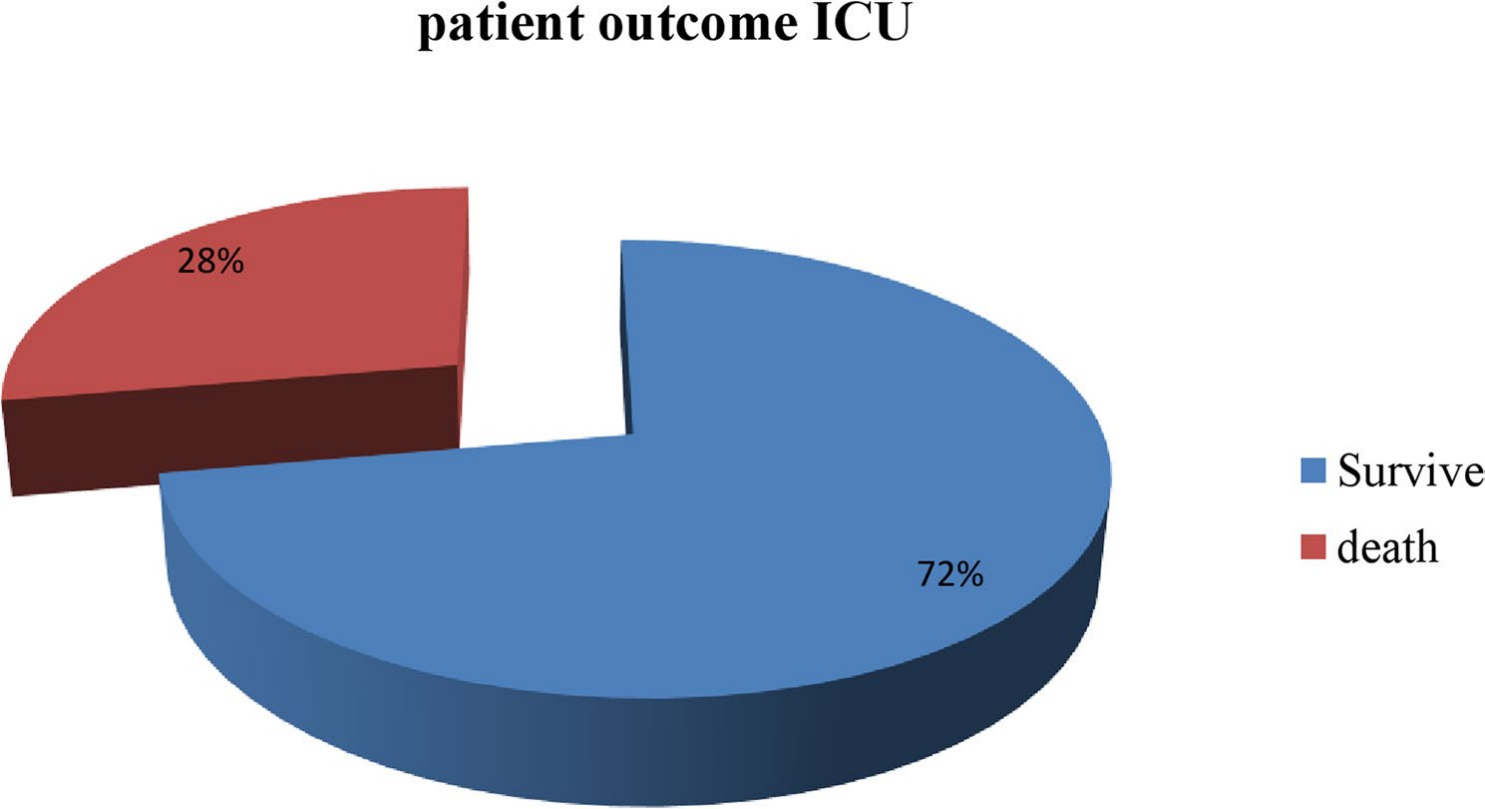
Discharge outcomes of adult patients admitted to the intensive care unit at Hawassa University Comprehensive Specialized Hospital, Ethiopia, 2024

As identified from this study, Outcomes during discharge among patients admitted to the ICU were 28.0% (95% CI: 23.5–32.5) deaths. This indicates that approximately three out of every ten adult patients admitted to the ICU died during their ICU stay (Fig. 1).

### Factors associated with the outcomes of patients admitted to the intensive care unit

Bivariate logistic analysis showed that Age, Frequency of admission, need for mechanical ventilation, Respiratory rate, length of ICU stay, and mental status at admission were significantly associated with the clinical outcome of ICU patients (*P* < 0.25). However, in a multivariate analysis, the associated risk factors of death were the need for mechanical ventilation, abnormal mental status at admission, and length of ICU stay.

Patients who stayed in the ICU for less than 4 days were two times more likely to die than those who stayed four days [AOR = 2.10 (1.25–3.53), CI = 95%].

There was a strong association between the level of consciousness and mortality. As a result, unconscious patients (GCS < 8) increase mortality by four times as compared with conscious patients [AOR = 4.46 (1.38–16.44), CI = 95%].

Patients who required mechanical ventilation had a fivefold increased risk of mortality compared to those who did not [AOR = 4.94 (1.49–21.8), CI = 95%] (Table 5).

**Table 5:** Bivariate and multivariate logistic regression analysis of factors associated with ICU mortality among adult patients at Hawassa University Comprehensive Specialized Hospital, Ethiopia (*n* = 401)

Variable		Patient Outcome		OR (95% CI)		*P*-value
		Death	Survive	COR(95%CI)	AOR(95%CI)	
Age	< 49	25 (22.5)	110(37.9)	**1**	1	
	50–59	47(42.3)	101 (34.8)	2.01(1.18–3.57)	0.85 (0.27–2.48.27.48)	0.80
	> 60	39 (35.1)	79 (27.2)	2.17(1.22–3.88)	0.60(0.18 − 2.00)	0.40
Length of ICU stay	< 4 days	51(45.9)	83 (28.6)	2.12 (1.35–3.39)	1.82 (1.25–3.53)	**0.00**
	≥ 4 days	60 (54.1)	207(71.4)	**1**	1	
Frequency of admission	New admission	101 (91.5)	276 (95.4)	**1**	1	
	Readmission	10 (9.0)	14 (4.8)	1.95(0.84–4.53)	1.74 (0.68–4.46)	0.246
RR	12–20	78 (70.3)	182(62.8)	**1**	1	
	> 20	33 (29.7)	108(37.2)	0.71 (0.45–1.14)	0.71(0.43–1.19.43.19)	0.71
GCS	Conscious	22 (19.8)	112 (38.6)	**1**	1	
	Confused	46 (41.4)	106(36.6)	2.21(1.25–3.92)	2.94(0.92–9.44.92.44)	0.07
	unconscious	43(38.7)	72(24.8)	3.04(1.68–5.50)	4.43(1.38–16.44)	**0.01**
Antibiotic	no	27 (24.8)	52 (17.9)	1.47(0.87–2.49.87.49)	1.18(0.68–2.15.68.15)	0.507
	yes	84(75.7)	238(82.1)	1	1	
MV	No	103 (92.8)	286 (98.6)	**1**	1	
	Yes	8 (7.2)	4 (1.4)	5.55(1.64–18.83)	4.94 (1.49–21.8)	**0.011**

*AOR*Adjusted odds ratio, *COR *crude odds ratio, *mv *Mechanical ventilation, *RR *Respiratory rate, *GCS*Conscious (GCS 13−15), Confused (9−12), Unconscious (3−8)

## Discussion

The purpose of this study was to assess clinical outcomes and factors associated with mortality among patients admitted to the intensive care unit (ICU) at Hawassa University Comprehensive Specialized Hospital (HUCSH). This retrospective study describes the need for mechanical ventilation, frequent impaired consciousness at admission, short ICU length of stay among non-survivors, and a considerable mortality rate.

The overall ICU mortality in this study was 28%, indicating that approximately three out of every ten adult patients admitted to the ICU died during their ICU stay. This mortality is lower than that of comparable studies conducted in Addis Ababa (39%) [11], Hossana (46.4%) [12], and Nigeria 43.5% [20]. This difference might be explained by variation in patient case mix, availability of critical-care resources, referral patterns, and timing of ICU admission. In resource-limited settings, delayed presentation and limited access to advanced supportive care continue to contribute to poor ICU outcomes.

However, when compared with high-income countries, the ICU mortality observed in this study remains higher than that reported in North America (9.3%), Oceania (10.3%), Europe (18.7%), and parts of Asia (13.7%) [21]. This persistent disparity reflects structural and systemic challenges in low- and middle-income countries, including delayed presentation, limited ICU bed capacity, shortages of trained intensivists and critical-care nurses, inconsistent availability of essential medications, and inadequate access to advanced organ-support technologies [22]. These factors collectively contribute to poorer outcomes despite intensive care unit admission.

This study revealed that patients admitted to the ICU and the number of deaths showed a shorter ICU length of stay (< 4 days) was significantly associated with mortality, which was supported by a study conducted in Kenya [6] and northern Ethiopia [13]. Early mortality often reflects the severe physiological instability of patients upon arrival. In resource-limited settings, patients are usually referred late, have inadequate initial resuscitation in emergency wards, lack inotropes, and have late access to mechanical ventilation. Many critically ill patients arrive in the ICU in irreversible shock or multiorgan failure, explaining higher mortality before 72–96 h [23].

The findings of this study reveal that patients who presented with an impaired level of consciousness at ICU admission were also independently associated with mortality. This study is consistent with studies conducted in Gondar Comprehensive and Specialized Hospital, Northwest Ethiopia [13] and Tanzania [8]. This result may be linked to the severity of the disease condition during admission. Altered mental status is related to severe decompensated disease, cerebral hypoperfusion due to sepsis, blood loss, poisoning, hypoxia, metabolic derangements, intracranial pathology, and shock-related cerebral hypoperfusion [24]. Besides this, patients with impaired consciousness are at increased risk of airway compromise, aspiration, secondary infections, and delayed recognition of clinical deterioration, all of which contribute to higher mortality in the ICU setting [25].

This study found that the need for mechanical ventilation was significantly associated with increased ICU mortality, which was similar to studies in Gondor in which the mechanical ventilator was an independent risk factor for death [13], and Kenya [26]. The possible explanation for this association could be related to the fact that mechanical ventilation was identified as an independent predictor of death. Mechanical ventilation is generally initiated in patients with severe respiratory failure, compromised airway protection, or hemodynamic instability, conditions that inherently carry a poor prognosis. Furthermore, in low- and middle-income countries (LMIC) ICUs, prolonged ventilation is frequently complicated by ventilator-associated pneumonia, sepsis, barotrauma, and limited access to lung-protective ventilation strategies, which further increase the risk of mortality [21].

## Conclusion

High mortality rates were recorded among the admitted adult patients within the intensive care unit of Hawassa University Comprehensive Specialized Hospital. Factors that were found to be associated with high mortality rates included the requirement for ventilatory support, low level of consciousness, and shorter stay within the intensive care unit. These results indicate that early deaths are associated with the severity of illness presented.

### Recommendations

Based on the study findings, the following recommendations are suggested:

Early identification and timely referral of critically ill patients, particularly those with altered mental status or respiratory failure, should be strengthened.

Better pre-ICU stabilization and post-ventilation patient surveillance could help minimize early death rates within the intensive care unit.

Improvement of critical care capacity, including necessary infrastructure, support staff, and critical care providers, has been advocated.

Future prospective studies involving multiple centers and using standardized variables for scoring the severity of illness are required to further identify predictors of in-hospital death in ICUs.

### Limitations of this study

There are some limitations to this study. First, it has been carried out in a way that it used hospital records, which could be subject to some incompleteness and misclassification. Second, severe symptoms of the disease, like ICU severity scores, could not be measured because they were not available. Third, this research work has been carried out in a single institution that might not be generalizable to other places. Lastly, this work has not included children, and it is relevant only to adults.

## Data Availability

We described all the relevant information in the manuscript, but the refined dataset can be obtained from the corresponding author upon reasonable request.

## Abbreviations

AOR: Adjusted odds ratio
ARDS: Acute respiratory distress syndrome
CI: Confident interval
COR: Crudes odds ratio
CHF: Congestive heart failure
DKA: Diabetic ketoacidosis
HUCSH: Hawassa University Comprehensive Specialized Hospital
HIV: Human immune-deficiency virus
MV: Mechanical ventilator
SPSS: Statistical Package for Social Science
TBI: Traumatic brain injury
ICU: Intensive care unit
